# Clinicopathological characteristics, survival outcomes, and genetic alterations of younger patients with gastric cancer: Results from the China National Cancer Center and cBioPortal datasets

**DOI:** 10.1002/cam4.4669

**Published:** 2022-03-22

**Authors:** Penghui Niu, Huang Huang, Lulu Zhao, Tongbo Wang, Xiaojie Zhang, Wanqing Wang, Yawei Zhang, Chunguang Guo, Dongbing Zhao, Yingtai Chen

**Affiliations:** ^1^ National Cancer Center/National Clinical Research Center for Cancer/Cancer Hospital Chinese Academy of Medical Sciences and Peking Union Medical College Beijing China

**Keywords:** clinicopathological characteristics, gastric cancer, genetic alterations, survival outcomes, younger patient

## Abstract

**Background:**

The survival outcomes of younger patients with gastric cancer (GC) have remained controversial. This study explores the clinicopathological characteristics, survival outcomes, and genetic alterations of younger and older patients with GC.

**Methods:**

Patients with GC were identified from the China National Cancer Center Gastric Cancer Database (NCCGCDB) during 1998–2018. Survival analysis was conducted using Kaplan–Meier estimates and Cox proportional hazards models. Sequencing datasets were enrolled from The Cancer Genome Atlas (TCGA) and Memorial Sloan–Kettering Cancer Center (MSKCC) databases.

**Results:**

A total of 1146 younger (<40 years of age) and 16,988 older (≥40 years of age) cases were included in the study. Younger patients had more poorly differentiated lesions than older patients (53.7% vs. 33.8%, respectively; *p* < 0.0001), and were more often pTNM stage IV (19.5% vs. 11.8%, respectively; *p* < 0.001). The 5‐year overall survival (OS) of patients from the NCCGCDB increased from 1998 to 2018. Younger patients with pTNM stage III had a lower survival rate than older patients (*p* = 0.014), while no differences by age were observed at other stages. The mutation frequency of the *LRP1B*, *GNAS*, *APC*, and *KMT2D* genes was higher for older than younger patients (*p* < 0.05 for all genes). While not significantly different, younger patients from the TCGA and MSKCC databases were more likely to have *CDH1*, *RHOA*, and *CTNNB1* gene mutations.

**Conclusions:**

A stable proportion and improved survival of younger patients were reported using NCCGCDB data. Younger patients with pTNM stage III had lower rates of survival than older patients. Distinct molecular characteristics were identified in younger GC patients which may partly explain the histopathology and prognosis specific to this subpopulation.

## Graphical abstract

Stable proportion of younger cases and noticeable survival improvements were reported in the China National Cancer Center. Younger patients with pTNM stage III had a worse survival than older.Furthermore, distinctive molecular characteristics were identified in younger GC patients, which might partly explain histopathological behaviors and prognosis of in this subpopulation.

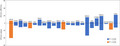

## 1 INTRODUCTION

Gastric cancer (GC) remains one of the most frequent digestive cancers and is the fourth leading cause of all cancer‐related death worldwide.^1^ More than 40% of GC cases and deaths occur in China, with an age‐standardized 5‐year survival rate of 35.1%.^2^, ^3^, ^4^ GC onset typically begins in patients ages 50–70 years, with younger patients only accounting for 2.0%–6.2% of cases.^5^, ^6^ Although GC incidence has declined in the general population, concern has been raised about stable or increasing GC rates among younger patients over the past few decades.^7^, ^8^, ^9^

GC in younger patients is associated with specific characteristics including female predominance, poorly differentiated lesions, and advanced‐stage diagnosis.^10^, ^11^, ^12^, ^13^ There are conflicting results on the survival outcomes of younger GC patients.^10^, ^11^, ^12^, ^13^, ^14^, ^15^ While some studies report that they have a longer survival time than older patients,^10^, ^11^ some studies show no difference in survival,^12^, ^13^ and others indicate that younger adults with GC have lower survival rates.^14^, ^15^ The increased incidence of GC in younger patients may be the result of its distinct pathology, with particular genetic alterations playing a critical role in disease progression and prognosis among different age groups.^16^, ^17^ However, information about the mutation profiles of younger GC patients is limited, and a comprehensive molecular profile of this patient population is necessary to better understand the molecular pathology of this disease.

This study sought to explore the clinicopathological characteristics and survival outcomes of younger and older patients in the China National Cancer Center Gastric Cancer Database (NCCGCDB) over the past 20 years, and create a reference for a larger population‐based study in China. Genetic alterations of younger patients were comprehensively investigated using the cBioPortal for Cancer Genomics database, informing the development of precision treatment for this subpopulation.

## 2 METHODS

### 2.1 Data source and patient selection

The study population was described in detail in previous studies.^18^, ^19^ In brief, all histologically confirmed incident cases of GC diagnosed between 1998 and 2018 were identified from the NCCGCDB. The NCCGCDB is a clinical gastric cancer database that was sourced from the China National Cancer Center. As a single large‐volume cohort, the NCCGCDB includes more than 18,000 patients from all regions of China that received a cancer diagnosis in the past 20 years. Enrollment criteria included age between 18 and 85 years, residence in China, and no cancer prior to GC diagnosis. All GC cases that met the inclusion criteria were divided into a younger (<40 years of age) and an older age group (≥40 years of age). The age cutoff for the younger group was created to remain consistent with most prior studies.^11^, ^20^ Clinical data abstracted from the NCCGCDB included patient demographics, clinicopathological characteristics, and survival variables. GC stage was assessed according to the eighth edition of the American Joint Committee on Cancer (AJCC) TNM staging system. The primary endpoints of the study were overall survival (OS) and progression‐free survival (PFS). Based on the time of histological diagnosis, changing trends of younger and older GC patients with GC were investigated during four consecutive time periods: 1998 to 2003 (period 1), 2003 to 2008 (period 2), 2008 to 2013 (period 3), and 2013 to 2018 (period 4). To further evaluate mutational differences between younger and older patients with gastric cancer, high‐volume genetic resources were obtained from the online cBioPortal for Cancer Genomics (http://www.cbioportal.org/).^19^, ^20^, ^21^ Eight gastric adenocarcinoma datasets from the TCGA and MSKCC databases met the inclusion criteria, and a total of 1098 GC cases with clear clinical data and genetic mutation information using next‐generation sequencing (NGS) were included in the comparative analysis.

### 2.2 Statistical analysis

A bar chart was plotted to evaluate the variation in tumor stage among younger and older patients from 1998 to 2018. Chi‐square analysis was performed to compare categorical variables between the two groups, while the Student's *t*‐test was used to evaluate continuous variables. The Kaplan–Meier method was used to calculate OS and PFS for the younger and older age groups, while the log‐rank test estimated the relevant survival discrepancy. Associations between risk factors and OS were investigated using univariate and multivariate Cox proportional hazard regression analysis, while the corresponding hazard ratio (HR) and 95% CI were generated. Covariates included in the final models were determined using stepwise selection with a minimized Akaike information criterion (AIC). The significance levels for adding and removing variables were 0.05 and 0.10, respectively. Statistical significance was set at two‐sided *p* values < 0.05, and all analyses in the study were conducted using SAS software v9.4 (SAS Institute, Inc.).

## 3 RESULTS

### 3.1 Demographic and clinicopathological features

A total of 1146 (6.3%) younger cases and 16,988 (93.7%) older cases from 1998 to 2018 were included in the study. Of all GC patients, 2035 were diagnosed in period 1, 3859 in period 2, 6054 in period 3, and 6150 in period 4 (Table 1). There were significant differences in gender, smoking, alcohol history, BMI, primary tumor location, differentiation, and pTNM stage between the younger and older groups (all *p* < 0.01). Distinct demographic disparities were found within the age groups of patients seen at the China National Cancer Center. Younger patients were predominantly female (50.1% vs. 21.6% for the older age group; *p* < 0.0001). Conversely, the older age group had a higher percentage of smokers (21.1% vs. 42.4% for the younger age group; *p* < 0.0001), alcohol drinkers (20.9% versus 34.5% for the younger age group; *p* < 0.0001), and overweight/obesity (BMI ≥ 23) (36.1% vs. 53.4% for the younger age group; *p* < 0.0001).

**TABLE 1:** Demographic and clinicopathological characteristics of the younger and older GC patient groups in four consecutive time periods (bidirectional cohort 1998–2018)

	Period 1 (1998–2003)		*p* Value	Period 2 (2004–2008)		*p* Value	Period 3 (2009–2013)		*p* Value	Period 4 (2014–2018)		*p* Value
	Younger group	Older group		Younger group	Older group		Younger group	Older group		Younger group	Older group	
	*n* (%)	*n* (%)		*n* (%)	*n* (%)		*n* (%)	*n* (%)		*n* (%)	*n* (%)	
Gender												
Male	83 (50.0%)	1367 (73.14%)		112 (46.1%)	2744 (75.1%)		209 (52.6%)	4321 (76.4%)		168 (49.4%)	4384 (75.5%)	
Female	83 (50.0%)	502 (26.86%)	<0.0001	131 (53.9%)	908 (24.9%)	<0.0001	188 (47.4%)	1336 (23.6%)	<0.0001	172 (50.6%)	1426 (24.5%)	<0.0001
Smoking history												
No	132 (79.5%)	1180 (63.1%)		195 (80.3%)	2242 (61.4%)		302 (76.1%)	3110 (55.0%)		241 (70.9%)	2813 (48.4%)	
Yes	33 (19.9%)	670 (35.9%)		39 (16.1%)	1247 (34.2%)		84 (21.2%)	2393 (42.3%)		86 (25.3%)	2896 (49.9%)	
Unknown	1 (0.6%)	19 (1.0%)	0.0001	9 (3.7%)	163 (4.5%)	<0.0001	11 (2.8%)	154 (2.7%)	<0.0001	13 (3.8%)	101 (1.7%)	<0.0001
Drinking history												
No	131 (78.9%)	1328 (71.1%)		206 (84.8%)	2562 (70.2%)		304 (76.6%)	3594 (63.5%)		230 (67.7%)	3190 (54.9%)	
Yes	34 (20.5%)	520 (27.8%)		27 (11.1%)	919 (25.2%)		81 (20.4%)	1908 (33.7%)		97 (28.5%)	2519 (43.4%)	
Unknown	1 (0.6%)	21 (1.1%)	0.095	10 (4.1%)	171 (4.7%)	<0.0001	12 (3.0%)	155 (2.7%)	<0.0001	13 (3.8%)	101 (1.7%)	<0.0001
BMI (kg/m^2^) at diagnosis												
<18.5	29 (17.5%)	124 (6.6%)		50 (20.6%)	264 (7.2%)		52 (13.1%)	336 (5.9%)		54 (15.9%)	283 (4.9%)	
18.5–22.9	73 (44.0%)	733 (39.2%)		109 (44.9%)	1346 (36.9%)		182 (45.8%)	2162 (38.2%)		143 (42.1%)	2101 (36.2%)	
23–27.4	48 (28.9%)	755 (40.4%)		53 (21.8%)	1388 (38.0%)		110 (27.7%)	2262 (40.0%)		98 (28.8%)	2574 (44.3%)	
≥27.5	9 (5.4%)	196 (10.5%)		17 (7.0%)	423 (11.6%)		39 (9.8%)	701 (12.4%)		40 (11.8%)	766 (13.2%)	
Unknown	7 (4.2%)	61 (3.3%)	<0.0001	14 (5.8%)	231 (6.3%)	<0.0001	14 (3.5%)	196 (3.5%)	<0.0001	5 (1.5%)	86 (1.5%)	<0.0001
Weight loss												
None	61 (36.8%)	615 (32.9%)		104 (42.8%)	1427 (39.1%)		241 (60.7%)	3238 (57.2%)		209 (61.5%)	3504 (60.3%)	
<10%	44 (26.5%)	461 (24.7%)		50 (20.6%)	842 (23.1%)		78 (19.7%)	1419 (25.1%)		74 (21.8%)	1531 (26.4%)	
≥10%	25 (15.1%)	191 (10.2%)		23 (9.5%)	339 (9.3%)		46 (11.6%)	547 (9.7%)		37 (10.9%)	557 (9.6%)	
Unknown	36 (22.7%)	602 (32.2%)	0.022	66 (27.2%)	1044 (28.6%)	0.66	32 (8.1%)	453 (8.0%)	0.086	20 (5.9%)	218 (3.8%)	0.072
*H. pylori* infection												
Negative	0 (0.0%)	3 (0.2%)		1 (0.4%)	51 (1.4%)		23 (5.8%)	397 (7.0%)		59 (17.4%)	1004 (17.3%)	
Positive	0 (0.0%)	11 (0.6%)		2 (0.8%)	42 (1.2%)		38 (9.6%)	522 (9.2%)		27 (7.9%)	524 (9.0%)	
Unknown	166 (100.0%)	1855 (99.3%)	1.00	240 (98.8%)	3559 (97.5%)	0.50	336 (84.6%)	4738 (83.8%)	0.64	254 (74.7%)	4282 (73.7%)	0.79
Primary tumor location												
Proximal	28 (16.9%)	836 (44.7%)		32 (13.2%)	1403 (38.4%)		65 (16.4%)	2164 (38.3%)		49 (14.4%)	2115 (36.4%)	
Distal	126 (75.9%)	898 (48.1%)		198 (81.5%)	1996 (54.7%)		296 (74.6%)	3082 (54.5%)		265 (77.9%)	3105 (53.4%)	
Overlapping lesions	6 (3.6%)	85 (4.6%)		3 (1.2%)	94 (2.6%)		19 (4.8%)	250 (4.4%)		15 (4.4%)	422 (7.3%)	
Unknown	6 (3.6%)	50 (2.7%)	<0.0001	10 (4.1%)	159 (4.4%)	<0.0001	17 (4.3%)	161 (2.9%)	<0.0001	11 (3.2%)	168 (2.9%)	<0.0001
Lauren classification												
Intestinal	0 (0.0%)	0 (0.0%)		0 (0.0%)	1 (0.0%)		16 (4.0%)	1073 (19.0%)		23 (6.8%)	1724 (29.7%)	
Diffuse	0 (0.0%)	1 (0.1%)		0 (0.0%)	1 (0.0%)		111 (28.0%)	942 (16.7%)		173 (50.9%)	1380 (23.8%)	
Mixed	0 (0.0%)	1 (0.1%)		0 (0.0%)	4 (0.1%)		25 (6.3%)	607 (10.7%)		47 (13.8%)	1115 (19.2%)	
Unknown	166 (100.0%)	1867 (99.9%)	1.00	243 (100.0%)	3646 (99.8%)	1.00	245 (61.7%)	3035 (53.7%)	<0.0001	97 (28.5%)	1591 (27.4%)	<0.0001
Borrmann classification												
Borrmann I	13 (7.8%)	210 (11.2%)		6 (2.5%)	316 (8.7%)		30 (7.6%)	537 (9.5%)		29 (8.5%)	587 (10.1%)	
Borrmann II	45 (27.1%)	750 (40.1%)		96 (39.5%)	1327 (36.3%)		134 (33.8%)	2115 (37.4%)		107 (31.5%)	2085 (35.9%)	
Borrmann III	52 (31.3%)	400 (21.4%)		52 (21.4%)	859 (23.5%)		87 (21.9%)	1304 (23.1%)		93 (27.4%)	1666 (28.7%)	
Borrmann IV	16 (9.6%)	116 (6.2%)		23 (9.5%)	306 (8.4%)		37 (9.3%)	316 (5.6%)		20 (5.9%)	250 (4.3%)	
Mixed	2 (1.2%)	7 (0.4%)		0 (0.0%)	9 (0.3%)		1 (0.3%)	12 (0.2%)		11 (3.2%)	150 (2.6%)	
Unknown	38 (22.9%)	386 (20.7%)	0.0010	66 (27.2%)	835 (22.9%)	0.014	108 (27.2%)	1373 (24.3%)	0.022	80 (23.5%)	1072 (18.5%)	0.090
Linitis plastica												
No	162 (97.6%)	1851 (99.0%)		232 (95.5%)	3474 (95.1%)		359 (90.4%)	5312 (93.9%)		321 (94.4%)	5565 (95.8%)	
Yes	4 (2.4%)	9 (0.5%)		1 (0.4%)	13 (0.4%)		13 (3.3%)	57 (1.0%)		11 (3.2%)	72 (1.2%)	
Unknown	0 (0.0%)	9 (0.5%)	0.028	10 (4.1%)	165 (4.5%)	0.95	25 (6.3%)	288 (5.1%)	0.0001	8 (2.4%)	173 (3.0%)	0.0068
Differentiation												
Well	1 (0.6%)	44 (2.4%)		0 (0.0%)	60 (1.6%)		4 (1.0%)	126 (2.2%)		1 (0.3%)	130 (2.2%)	
Moderate	21 (12.7%)	658 (35.2%)		26 (10.7%)	1162 (31.8%)		34 (8.6%)	1882 (33.3%)		42 (12.4%)	2167 (37.3%)	
Poor	87 (52.4%)	575 (30.8%)		116 (47.7%)	1179 (32.3%)		216 (54.4%)	1931 (34.1%)		196 (57.7%)	2054 (35.4%)	
Undifferentiated	0 (0.0%)	1 (0.1%)		0 (0.0%)	0 (0.0%)		0 (0.0%)	2 (0.0%)		0 (0.0%)	1 (0.0%)	
Unknown	57 (34.3%)	591 (31.6%)	<0.0001	101 (41.6%)	1251 (34.3%)	<0.0001	143 (36.0%)	1716 (30.3%)	<0.0001	101 (29.7%)	1458 (25.1%)	<0.0001
HER2 score												
0 (−)	1 (0.6%)	0 (0.0%)		3 (1.2%)	19 (0.5%)		90 (22.7%)	1231 (21.8%)		120 (35.3%)	2020 (34.8%)	
1 (+)	0 (0.0%)	1 (0.1%)		0 (0.0%)	3 (0.1%)		96 (24.2%)	1291 (22.8%)		107 (31.5%)	1523 (26.2%)	
2 (++)	0 (0.0%)	1 (0.1%)		0 (0.0%)	3 (0.1%)		27 (6.8%)	531 (9.4%)		19 (5.6%)	673 (11.6%)	
3 (+++)	0 (0.0%)	0 (0.0%)		0 (0.0%)	1 (0.0%)		12 (3.0%)	293 (5.2%)		7 (2.1%)	288 (5.0%)	
Unknown	165 (99.4%)	1867 (99.9%)	0.23	240 (98.8%)	3626 (99.3%)	0.46	172 (43.3%)	2311 (40.9%)	0.13	87 (25.6%)	1306 (22.5%)	0.0004
Pathologic T‐stage												
T0 + Tis	0 (0.0%)	1 (0.1%)		2 (0.8%)	13 (0.4%)		1 (0.3%)	20 (0.4%)		0 (0.0%)	24 (0.4%)	
T1	17 (10.2%)	159 (8.5%)		37 (15.2%)	357 (9.8%)		59 (14.9%)	796 (14.1%)		81 (23.8%)	1269 (21.8%)	
T2	13 (7.8%)	135 (7.2%)		15 (6.2%)	248 (6.8%)		30 (7.6%)	491 (8.7%)		36 (10.6%)	554 (9.5%)	
T3	2 (1.2%)	13 (0.7%)		22 (9.1%)	311 (8.5%)		80 (20.2%)	1712 (30.3%)		39 (11.5%)	1159 (20.0%)	
T4	98 (59.0%)	1156 (61.9%)		110 (45.3%)	1903 (52.1%)		116 (29.2%)	1374 (24.3%)		114 (33.5%)	1654 (28.5%)	
TX	36 (21.7%)	405 (21.7%)	0.74	57 (23.5%)	820 (22.5%)	0.066	111 (28.0%)	1264 (22.3%)	0.0004	70 (20.6%)	1150 (19.8%)	0.0042
Pathologic N‐stage												
N0	27 (16.3%)	418 (22.4%)		62 (25.5%)	830 (22.7%)		97 (24.4%)	1518 (26.8%)		110 (32.4%)	1893 (32.6%)	
N1	21 (12.7%)	273 (14.6%)		31 (12.8%)	520 (14.2%)		41 (10.3%)	775 (13.7%)		31 (9.1%)	729 (12.6%)	
N2	29 (17.5%)	310 (16.6%)		24 (9.9%)	510 (14.0%)		52 (13.1%)	797 (14.1%)		55 (16.2%)	766 (13.2%)	
N3	50 (30.1%)	459 (24.6%)		64 (25.3%)	888 (24.3%)		91 (22.9%)	1222 (21.6%)		70 (20.6%)	1195 (20.6%)	
NX	39 (23.5%)	409 (21.9%)	0.27	62 (25.5%)	904 (24.8%)	0.36	116 (29.2%)	1345 (23.8%)	0.060	74 (21.8%)	1227 (21.1%)	0.26
Pathologic M‐stage												
M0	122 (73.5%)	1563 (83.6%)		178 (73.3%)	2939 (80.5%)		284 (71.5%)	4517 (79.9%)		266 (78.2%)	4882 (84.0%)	
M1	40 (24.1%)	261 (14.0%)		48 (19.8%)	430 (11.8%)		81 (20.4%)	714 (12.6%)		54 (15.9%)	601 (10.3%)	
MX	4 (2.4%)	45 (2.4%)	0.0020	17 (7.0%)	283 (7.8%)	0.0012	32 (8.1%)	426 (7.5%)	<0.0001	20 (5.9%)	327 (5.6%)	0.0051
pTNM stage												
0	0 (0.0%)	1 (0.1%)		2 (0.8%)	12 (0.3%)		1 (0.3%)	20 (0.4%)		0 (0.0%)	19 (0.3%)	
I	17 (10.2%)	210 (11.2%)		42 (17.3%)	470 (12.9%)		71 (17.9%)	1010 (17.9%)		94 (27.7%)	1422 (24.5%)	
II	10 (6.0%)	63 (3.4%)		24 (9.9%)	279 (7.6%)		40 (10.1%)	948 (16.8%)		34 (10.0%)	935 (16.1%)	
III	85 (51.2%)	1103 (59.0%)		99 (40.7%)	1895 (51.9%)		153 (38.5%)	2182 (38.6%)		128 (37.7%)	2048 (35.3%)	
IV	40 (24.1%)	261 (14.0%)		48 (19.8%)	430 (11.8%)		81 (20.4%)	714 (12.6%)		54 (15.9%)	601 (10.3%)	
Unknown	14 (8.4%)	231 (12.4%)	0.0039	28 (11.5%)	566 (15.5%)	<0.0001	51 (12.9%)	783 (13.8%)	<0.0001	30 (8.8%)	785 (13.5%)	0.0001
Lymphatic invasion												
No	76 (45.8%)	986 (52.8%)		123 (50.6%)	1777 (48.7%)		183 (46.1%)	2830 (50.0%)		152 (44.7%)	2384 (41.0%)	
Yes	36 (21.7%)	357 (19.1%)		34 (14.0%)	749 (20.5%)		75 (18.9%)	1182 (20.9%)		82 (24.1%)	1959 (33.7%)	
Unknown	54 (32.5%)	526 (28.1%)	0.23	86 (35.4%)	1126 (30.8%)	0.039	139 (35.0%)	1645 (29.1%)	0.043	106 (31.2%)	1467 (25.3%)	0.0008
Vascular invasion												
No	76 (45.8%)	988 (52.9%)		124 (51.0%)	1776 (48.6%)		183 (46.1%)	2828 (50.0%)		152 (44.7%)	2375 (40.9%)	
Yes	36 (21.7%)	354 (18.9%)		34 (14.0%)	742 (20.3%)		74 (18.6%)	1185 (21.0%)		82 (24.1%)	1973 (34.0%)	
Unknown	54 (32.5%)	527 (28.2%)	0.22	85 (35.0%)	1134 (31.1%)	0.051	140 (35.3%)	1644 (29.1%)	0.031	106 (31.2%)	1462 (25.2%)	0.0006
Nerve invasion												
No	110 (66.3%)	1330 (71.2%)		153 (63.0%)	2427 (66.5%)		186 (46.9%)	3094 (54.7%)		117 (34.4%)	2120 (36.5%)	
Yes	2 (1.2%)	13 (0.7%)		5 (2.1%)	100 (2.7%)		69 (17.4%)	917 (16.2%)		122 (35.9%)	2321 (40.0%)	
Unknown	54 (32.5%)	526 (28.1%)	0.35	85 (35.0%)	1125 (30.8%)	0.35	142 (35.8%)	1646 (29.1%)	0.0064	101 (29.7%)	1369 (23.6%)	0.034
Therapeutic regimen												
Surgery only	3 (1.8%)	99 (5.3%)		12 (4.9%)	149 (4.1%)		19 (4.8%)	347 (6.1%)		55 (16.2%)	871 (15.0%)	
Multimodality treatment	54 (32.5%)	452 (24.2%)		72 (29.6%)	908 (24.9%)		166 (41.8%)	1800 (31.8%)		149 (43.8%)	2052 (35.3%)	
Unknown	109 (65.7%)	1318 (70.5%)	0.014	159 (65.4%)	2595 (71.1%)	0.18	212 (53.4%)	3510 (62.1%)	0.0002	136 (40.0%)	2887 (49.7%)	0.0016
Surgical margin												
Negative	120 (72.3%)	1355 (72.5%)		173 (71.2%)	2650 (72.6%)		265 (66.8%)	4110 (72.7%)		251 (73.8%)	4417 (76.0%)	
Positive	11 (6.6%)	110 (5.9%)	0.92	4 (1.7%)	103 (2.8%)	0.41	8 (2.0%)	106 (1.9%)	0.037	7 (2.1%)	92 (1.6%)	0.58

GC tumors in younger patients were more often distally located (77.2% vs. 53.5% for the older age group; *p* < 0.0001), Borrmann IV (8.4% vs. 5.8% for the older age group; *p* < 0.05), poorly differentiated (53.7% vs. 33.8% for the older age group; *p* < 0.0001), and pTNM stage IV (19.5% vs. 11.8% for the older age group; *p* < 0.001). Older patients more often had tumors with negative surgical margins than younger patients, but this was not statistically significant during any time periods (all *p* ≥ 0.05). Of patients in periods 3 and 4, younger GC patients were more likely to present with diffuse classification (*p* < 0.0001), and have lymphatic and vascular invasion (*p* = 0.043, *p* = 0.0008, *p* = 0.031, *p* = 0.0006, respectively). In periods 1, 3, and 4, younger GC patients also demonstrated better acceptance of multimodality treatment (32.5% vs. 24.2%, *p* = 0.014; 41.8% vs. 31.8%, *p* < 0.001; 43.8% vs. 35.3%, *p* = 0.0016, for younger and older patients, respectively).

**FIGURE 1:**
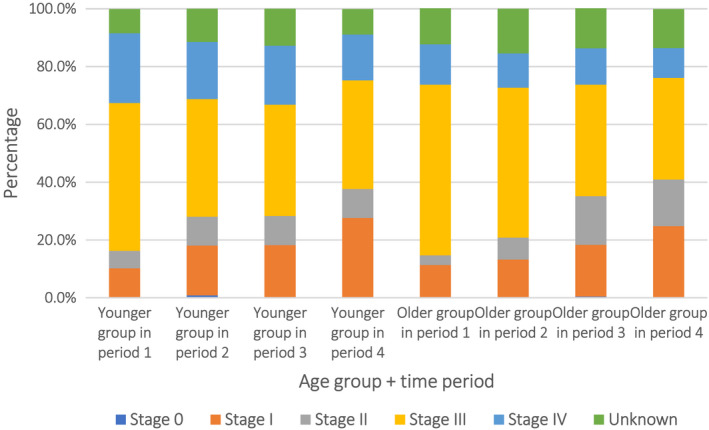
Changing trends of the proportion of younger and older groups by period and pTNM tumor stage

Changes in the clinicopathological features of younger GC patients over the past 20 years were also investigated. The median proportion of patients in the younger group was 6.2% (range 5.5%–8.2%). Linear regression showed no change in the proportion of younger patients in periods 2 to 4 (*p* = 0.053). The proportion of HER2 scores of 0(−) and 1(+) increased from period 3 to period 4 (22.7% to 35.3% for a HER2 score of 0(−) and 24.2% to 31.5% for a HER2 score of 1(+)), while scores of 2(++) and 3(+++) decreased (6.8% to 5.6% and 3.0% to 2.1%, respectively). The percentage of pTNM tumor stages I and II in the younger group increased gradually with time (from 10.2% in period 1 to 22.7% in period 4 and from 6.0% in period 1 to 10.0% in period 4), while GC in pTNM stages III and IV decreased (from 51.2% in period 1 to 37.7% in period 4, and from 24.1% in period 1 to 15.9% in period 4, respectively) (Figure 1). The percentages of younger patients undergoing surgery and those receiving multimodality treatment increased from periods 1 to 4 (1.8% to 16.2% and 32.5% to 42.8%, respectively). There was a significant decline in positive surgical margins among younger patients over the 20‐year time period (from 6.6% in period 1 to 2.1% in period 4).

### 3.2 OS and PFS of younger patients with GC

**FIGURE 2:**
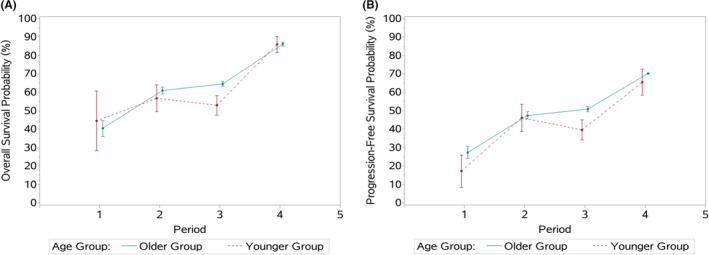
Survival trends between younger and older groups during 20 years. (A) The 5‐year overall survival. (B) The 5‐year progression‐free survival

The changing trends of OS and PFS in younger and older age groups are shown in Table 2 and Figure 2. A noticeable rise in GC survival was demonstrated at the Cancer Center over the 20‐year time period. The 5‐year OS in younger and older group increased from 44.4% (95%CI: 28.2%–60.7%) to 86.0% (95%CI: 81.7%–90.4%) and from 40.4% (95%CI: 36.0%–44.8%) to 86.3% (95%CI: 85.2%–87.4%), respectively. After stratifying by pTNM stage, the increase in survival of younger cases was pronounced in stages III and IV (from 31.3% in period 1 to 83.6% in period 4 and from 0% in period 1 to 55.5% in period 4, respectively). Moreover, the 5‐year PFS of younger patients increased from 17.1% (95% CI: 8.3%–26.0%) in period 1 to 65.5% (95% CI: 58.4%–72.6%) in period 4, with an obvious dominance of pTNM stage III (8.1%– 62%).

**TABLE 2:** The 5‐year overall and progression‐free survival rates by cancer stages (bidirectional cohort 1998–2018)

Survival analysis	Total, %(95% Cl)	pTNM stage I, %(95% Cl)	pTNM stage II, %(95% Cl)	pTNM stage III, %(95% Cl)	pTNM stage IV, %(95% Cl)
Total					
Overall survival					
Total (*n* = 13,533)	68.1 (67.2–68.9)	96.6 (95.8–97.3)	85.1 (83.3–86.8)	61.8 (60.4–63.2)	19.0 (16.3–21.8)
Younger (*n* = 837)	61.8 (58.2–65.4)	99.5 (98.5–100.0)	80.6 (71.6–89.5)	55.1 (49.0–61.2)	16.2 (8.4–24.0)
Older (*n* = 12,696)	68.5 (67.6–69.4)	96.3 (95.5–97.1)	85.3 (83.5–87.0)	62.3 (60.8–63.7)	19.4 (16.5–22.4)
Progression‐free survival					
Total (*n* = 12,728)	53.4 (52.4–54.3)	90.4 (89.1–91.7)	75.4 (73.2–77.5)	46.8 (45.4–48.2)	8.5 (6.6–10.4)
Younger (*n* = 856)	46.0 (42.4–49.6)	95.6 (92.4–98.8)	63.1 (52.2–74.1)	36.3 (30.7–42.0)	8.7 (3.6–13.8)
Older (*n* = 11,872)	53.9 (53.0–54.9)	89.9 (88.6–91.3)	76.0 (73.9–78.2)	47.5 (46.0–49.0)	8.4 (6.3–10.4)
Period 1					
Overall survival					
Total (*n* = 511)	40.7 (36.4–45.0)	94.0 (88.9–99.1)	78.3 (61.4–95.1)	32.4 (26.9–37.8)	4.8 (0.0–10.2)
Younger (*n* = 36)	44.4 (28.2–60.7)	100.0 (100.0–100.0)	100.0 (100.0–100.0)	31.3 (8.5–54.0)	0.0 (0.0–0.0)
Older (*n* = 475)	40.4 (36.0–44.8)	93.3 (87.7–99.0)	75.0 (56.0–94.0)	32.5 (26.8–38.1)	5.4 (0.0–11.3)
Progression‐free survival					
Total (*n* = 794)	26.5 (23.4–29.5)	79.8 (71.9–87.7)	42.4 (25.6–59.3)	21.7 (17.9–25.4)	2.2 (0.0–5.1)
Younger (*n* = 70)	17.1 (8.3–26.0)	88.9 (68.3–100.0)	16.7 (0.0–46.5)	8.1 (0.0–16.9)	0.0 (0.0–0.0)
Older (*n* = 724)	27.4 (24.1–30.6)	78.9 (70.5–87.3)	48.2 (29.3–67.0)	22.8 (18.8–26.8)	2.4 (0.0–5.7)
Period 2					
Overall survival					
Total (*n* = 2801)	60.7 (58.9–62.5)	93.9 (91.7–96.2)	77.2 (72.0–82.4)	57.5 (54.9–60.1)	12.2 (8.0–16.3)
Younger (*n* = 178)	56.7 (49.5–64.0)	100.0 (100.0–100.0)	66.7 (46.5–86.8)	45.7 (34.0–5.74)	16.7 (3.3–30.0)
Older (*n* = 2623)	60.9 (59.1–62.8)	93.4 (90.9–95.8)	78.2 (72.8–83.5)	58.2 (55.5–60.8)	11.5 (7.2–15.9)
Progression‐free survival					
Total (*n* = 2561)	47.3 (45.4–49.2)	87.8 (84.5–91.0)	67.9 (62.0–73.7)	45.4 (42.7–48.0)	9.8 (6.1–13.4)
Younger (*n* = 176)	46.0 (38.7–53.4)	94.3 (86.6–100.0)	52.2 (31.7–72.6)	37.3 (26.4–48.3)	16.1 (3.2–29.1)
Older (n = 2385)	47.4 (45.4–49.4)	87.1 (83.6–90.6)	69.5 (63.5–75.5)	45.9 (43.1–48.6)	8.9 (5.2–12.6)
Period 3					
Overall survival					
Total (*n* = 5211)	63.8 (62.5–65.1)	96.6 (95.5–97.7)	83.3 (80.8–85.7)	58.3 (56.2–60.5)	9.4 (6.9–11.9)
Younger (*n* = 331)	52.9 (47.5–58.2)	98.6 (95.7–100.0)	80.0 (66.8–93.2)	49.2 (40.2–58.1)	6.7 (0.4–13.0)
Older (*n* = 4880)	64.6 (63.2–65.9)	96.4 (95.3–97.6)	83.4 (80.9–85.8)	58.9 (56.7–61.1)	9.8 (7.0–12.5)
Progression‐free survival					
Total (*n* = 4635)	50.1 (48.6–51.5)	91.3 (89.4–93.2)	74.4 (71.4–77.5)	43.7 (41.4–45.9)	4.3 (2.6–6.0)
Younger (*n* = 318)	39.6 (34.2–45.0)	96.4 (91.6–100.0)	71.9 (56.3–87.5)	31.7 (23.5–39.9)	5.9 (0.3–11.5)
Older (*n* = 4317)	50.9 (49.4–52.3)	90.9 (88.9–92.9)	74.5 (71.5–77.6)	44.5 (42.2–46.9)	4.1 (2.4–5.9)
Period 4					
Overall survival					
Total (*n* = 5010)	86.3 (85.2–87.3)	98.7 (98.0–99.4)	94.1 (92.3–95.9)	83.5 (81.5–85.5)	53.0 (47.0–59.0)
Younger (*n* = 292)	86.0 (81.7–90.4)	100.0 (100.0–100.0)	96.9 (90.8–100.0)	83.6 (75.8–91.4)	55.5 (37.5–73.5)
Older (*n* = 4718)	86.3 (85.2–87.4)	98.6 (97.9–99.3)	94.0 (92.1–95.8)	83.5 (81.4–85.6)	52.8 (46.5–59.1)
Progression‐free survival					
Total (*n* = 4738)	69.8 (67.9–71.7)	92.2 (89.4–94.9)	84.8 (81.9–87.7)	66.5 (63.3–69.6)	6.8 (0.3–13.4)
Younger (*n* = 292)	65.5 (58.4–72.6)	95.3 (88.6–100.0)	80.3 (60.7–99.9)	62.0 (50.9–73.1)	0.0 (0.0–0.0)
Older (*n* = 4446)	70.1 (68.1–72.0)	91.9 (89.0–94.9)	85.0 (82.1–87.9)	66.8 (63.5–70.0)	7.7 (0.4–15.1)

**FIGURE 3:**
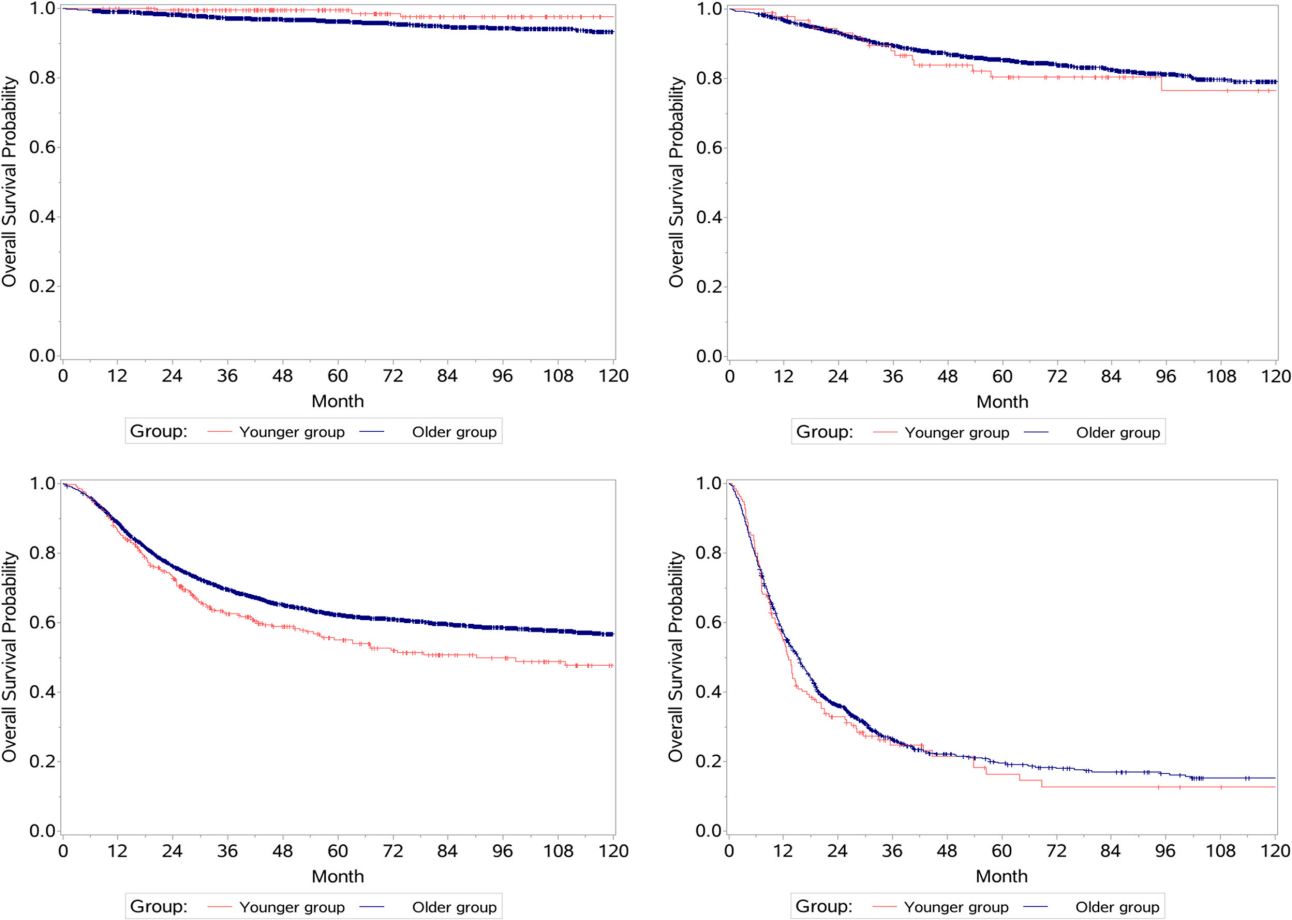
Kaplan–Meier survival curves of gastric cancer patients between YG and OG. (A) Kaplan–Meier survival curves of patients in pTNM stage I. (B) Kaplan–Meier survival curves of patients in pTNM stage II. (C) Kaplan–Meier survival curves of patients in pTNM stage III. (D) Kaplan–Meier survival curves of patients in pTNM stage IV

The Kaplan–Meier curves for OS in the younger and older groups are shown in Figure 3. The analysis showed that younger patients with stage III GC had a worse survival outcome (*p* = 0.0095) while those with stage I had a better prognosis (*p* = 0.03). However, no significant differences were found by age for stages II and IV (*p* = 0.60 and *p* = 0.37, respectively).

### 3.3 Prognostic factors in univariate and multivariate analyses

Univariate and multivariate analyses were performed to investigate significant factors impacting survival outcomes in younger GC patients. GC survival was significantly different based on the following parameters: the period of diagnosis, drinking consumption, overweight/obesity (BMI ≥23, <27.4), weight loss ≥10%, *H. pylori* infection, distal tumor location, Borrmann IV, Linitis plastica, poor differentiation, pTNM stage II, III, and IV, vascular invasion, and surgical margin (all *p* < 0.05) (Table 3). Among younger patients, prognosis factors only involved diagnosis period 4 (*p* < 0.0001), distal tumor location (*p* = 0.0007), Linitis plastica (*p* = 0.024), pTNM stage III (*p* < 0.0001), pTNM stage IV (*p* < 0.0001), and surgical margin (*p* = 0.011) (Table S1).

**TABLE 3:** Univariate and multivariate survival analysis of predictors associated with overall survival of patients with gastric cancer (bidirectional cohort 1998–2018)

Prognostic factors	Univariate analysis			Multivariate analysis		
	Hazard ratio	95% CI	*p* Value	Hazard ratio	95% CI	*p* Value
Period of diagnosis						
Period 1 (1998–2003)	1.00			1.00		
Period 2 (2004–2008)	0.54	0.48–0.61	<0.0001	0.55	0.49–0.62	<0.0001
Period 3 (2009–2013)	0.42	0.42–0.53	<0.0001	0.60	0.53–0.69	<0.0001
Period 4 (2014–2018)	0.18	0.16–0.21	<0.0001	0.24	0.20–0.28	<0.0001
Gender						
Male	1.00					
Female	1.06	0.99–1.14	0.091			
Smoking history						
No	1.00					
Yes	0.90	0.84–0.96	0.0009			
Unknown	1.06	0.89–1.25	0.54			
Drinking history						
No	1.00			1.00		
Yes	0.88	0.82–0.94	0.0001	0.93	0.87–1.00	0.045
Unknown	1.06	0.89–1.25	0.52	0.86	0.70–1.07	0.18
BMI (kg/m^2^) at diagnosis						
<18.5	1.17	1.04–1.32	0.0080	1.05	0.93–1.19	0.42
18.5–22.9	1.00			1.00		
23–27.4	0.80	0.74–0.85	<0.0001	0.93	0.86–1.00	0.036
≥27.5	0.75	0.68–0.83	<0.0001	0.94	0.85–1.05	0.25
Unknown	1.51	1.31–1.74	<0.0001	1.14	0.96–1.35	0.14
Weight loss						
None	1.00			1.00		
<10%	1.38	1.28–1.48	<0.0001	1.05	0.97–1.13	0.22
≥10%	2.14	1.95–2.35	<0.0001	1.22	1.11–1.35	<0.0001
Unknown	1.82	1.67–1.99	<0.0001	1.07	0.97–1.19	0.16
*H. pylori* infection						
Negative	1.00			1.00		
Positive	1.64	1.32–2.05	<0.0001	1.29	1.03–1.61	0.024
Unknown	3.09	2.62–3.66	<0.0001	1.54	1.30–1.83	<0.0001
Primary tumor location						
Proximal	1.00			1.00		
Distal	0.87	0.81–0.92	<0.0001	0.93	0.87–1.00	0.038
Overlapping lesions	1.56	1.36–1.78	<0.0001	1.10	0.96–1.27	0.18
Unknown	1.20	1.02–1.40	0.026	0.65	0.53–0.80	<0.0001
Lauren classification						
Intestinal	1.00			1.00		
Diffuse	1.83	1.58–2.12	<0.0001	1.17	0.99–1.38	0.069
Mixed	1.36	1.14–1.61	0.0005	0.97	0.81–1.15	0.70
Unknown	3.75	3.33–4.23	<0.0001	1.09	0.93–1.27	0.30
Borrmann classification						
Borrmann I	1.00			1.00		
Borrmann II	0.94	0.83–1.07	0.37	0.90	0.79–1.02	0.089
Borrmann III	1.17	1.03–1.33	0.019	0.97	0.85–1.11	0.68
Borrmann IV	2.48	2.13–2.88	<0.0001	1.40	1.19–1.65	<0.0001
Mixed	0.42	0.25–0.70	0.0009	0.97	0.57–1.63	0.90
Unknown	2.50	2.21–2.84	<0.0001	1.06	0.93–1.21	0.42
Linitis plastica						
No	1.00			1.00		
Yes	2.33	1.84–2.94	<0.0001	1.29	1.01–1.64	0.044
Unknown	1.51	1.33–1.71	<0.0001	0.85	0.72–0.99	0.042
Differentiation						
Well	1.00			1.00		
Moderate	3.10	2.03–4.74	<0.0001	1.45	0.94–2.22	0.090
Poor	4.48	2.94–6.83	<0.0001	1.56	1.02–2.40	0.041
Undifferentiated	0.001	0.001–999.999	0.91	0.000	0.000–999.999	0.90
Unknown	9.73	6.39–14.81	<0.0001	1.59	1.02–2.48	0.042
HER2 score						
0 (−)	1.00			1.00		
1 (+)	0.84	0.74–0.96	0.0082	0.96	0.85–1.09	0.54
2 (++)	0.71	0.59–0.84	0.0001	0.90	0.75–1.08	0.27
3 (+++)	1.05	0.85–1.29	0.68	1.08	0.87–1.33	0.51
Unknown	2.41	2.20–2.64	<0.0001	1.21	1.06–1.37	0.0042
Pathologic T‐stage						
T0 + Tis	2.67	1.17–6.08	0.020	1.38	0.19–10.14	0.75
T1	1.00			1.00		
T2	2.72	2.10–3.51	<0.0001	1.44	1.07–1.95	0.017
T3	7.01	5.70–8.62	<0.0001	2.10	1.51–2.92	<0.0001
T4	11.29	9.24–13.80	<0.0001	2.55	1.82–3.57	<0.0001
TX	19.07	15.58–23.33	<0.0001	2.50	1.72–3.62	<0.0001
Pathologic N‐stage						
N0	1.00			1.00		
N1	2.28	1.98–2.63	<0.0001	1.27	1.09–1.49	0.0020
N2	3.52	3.09–4.00	<0.0001	1.74	1.48–2.04	<0.0001
N3	6.85	6.11–7.67	<0.0001	2.82	2.41–3.29	<0.0001
NX	8.72	7.79–9.75	<0.0001	1.74	1.35–2.26	<0.0001
Pathologic M‐stage						
M0	1.00					
M1	5.76	5.34–6.22	<0.0001			
MX	1.98	1.78–2.20	<0.0001			
pTNM stage						
0	2.53	1.04–6.19	0.042	1.76	0.20–15.49	0.61
I	1.00			1.00		
II	3.70	2.99–4.57	<0.0001	1.69	1.23–2.32	0.0012
III	10.42	8.66–12.54	<0.0001	1.85	1.29–2.66	0.0009
IV	39.04	32.23–47.29	<0.0001	5.04	3.42–7.42	<0.0001
Unknown	13.19	10.88–15.99	<0.0001	1.80	1.21–2.68	0.0036
Lymphatic invasion						
No	1.00					
Yes	1.76	1.62–1.92	<0.0001			
Unknown	3.73	3.47–4.00	<0.0001			
Vascular invasion						
No	1.00			1.00		
Yes	1.77	1.63–1.92	<0.0001	1.17	1.07–1.28	0.0006
Unknown	3.74	3.48–4.01	<0.0001	1.17	1.00–1.38	0.054
Nerve invasion						
No	1.00					
Yes	1.06	0.96–1.16	0.23			
Unknown	3.15	2.95–3.37	<0.0001			
Therapeutic regimen						
Surgery only	1.00			1.00		
Multimodality treatment	3.73	3.04–4.58	<0.0001	1.09	0.88–1.34	0.44
Unknown	5.67	4.64–6.93	<0.0001	1.28	1.04–1.57	0.022
Surgical margin						
Negative	1.00			1.00		
Positive	2.47	2.08–2.94	<0.0001	1.24	1.04–1.48	0.015
Unknown	3.10	2.91–3.30	<0.0001	1.28	1.10–1.50	0.0020
Age group						
Younger group (<40 years)	1.00			1.00		
Older group (≥40 years)	0.80	0.71–0.90	0.0001	0.92	0.82–1.04	0.20

*Note*: The final model was built using stepwise selection with minimized AIC, and the covariates included in the final models were selected using the stepwise selection method, with a significance level for adding variables of 0.05 and a significance level for removing variables of 0.10.

In the univariate analysis, older GC patients had better survival outcomes than younger patients (HR = 0.80, 95% CI: 0.71–0.90, *p* = 0.0001). After stratification by pTNM stage, younger patients had a better stage I prognosis (*p* = 0.04) and a worse stage III prognosis (*p* < 0.01) than older patients (Table S2). However, multivariate analysis demonstrated that younger age was not an independent factor for poor survival outcomes (*p* = 0.20). In subdivided pTNM stages, younger patients with stage III tumors had worse survival outcomes (*p* = 0.014), but there were no statistically significant differences by age for stages I, II, and IV (*p* = 0.074, 0.59, and 0.76, respectively).

### 3.4 Genetic alterations of younger patients with GC

**FIGURE 4:**
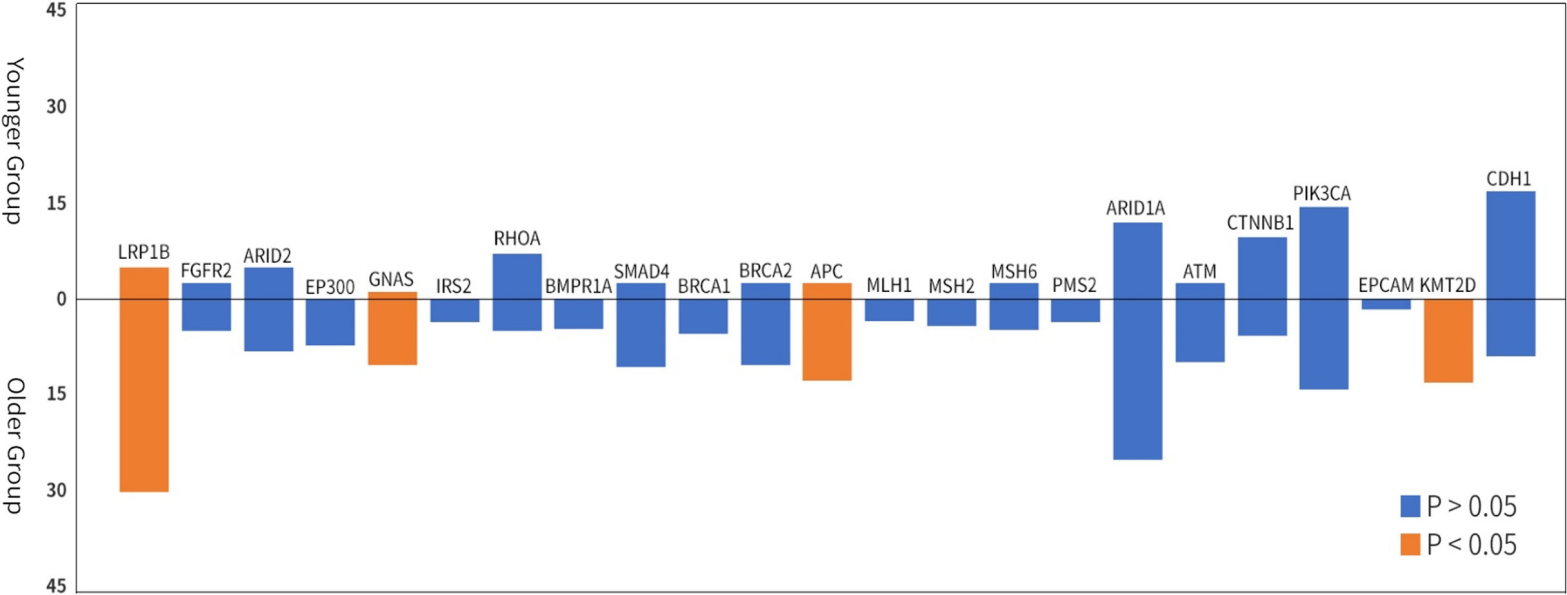
Comparison of mutation frequencies between younger and older groups from the TCGA and MSKCC databases

A high‐volume analysis of the relationship between genetic alterations and age groups was performed using TCGA and MSKCC data (Figure 4 and Figure S1). GC tissues from older patients had more *LRP1B* (30% vs. 9%, *p* < 0.05)*, KMT2D* (13% vs. 0%, *p* < 0.05), *APC* (13% vs. 2%, *p* < 0.05), and *GNAS* mutations (11% vs. 1%, *p* < 0.05) than GC tissues from younger patients. While not significant, younger patients had more *CDH1* (17% vs. 9%), *RHOA* (7% vs. 5%), and *CTNNB1* mutations (9% vs. 6%) than older patients. Of the eight pooled gastric adenocarcinoma datasets, high‐TMB was closely associated with older age at diagnosis (*p* < 0.0001) (Figure S2).

## 4 DISCUSSION

This study provides a comprehensive analysis of younger GC patients at both the clinical and molecular levels. A primary finding was the stable proportion and significant survival rate of younger patients seen in the China National Cancer Center from 1988 to 2018. Younger age was an independent prognostic factor for poor survival of pTNM stage III patients, while there were no differences by age in the survival rates of patients with stages I, II, or IV. The higher mutation frequency of *LRP1B, KMT2D*, *APC*, and *GNAS* genes in older GC patients may partially explain the age differences in histopathology and disease prognosis.

This study is consistent with prior studies,^10^, ^11^, ^12^, ^13^, ^14^, ^15^, ^22^, ^23^ indicating distinct clinical features among younger patients, including a higher proportion of females, poor tumor differentiation, and advanced tumor stage at diagnosis. While the mechanism for female predominance among younger patients remains unclear, some studies have suggested that hormonal factors may play a role.^24^, ^25^ In addition to *H. pylori* infection^26^ and some genetic distinctions,^27^ non‐specific symptoms and a delay in diagnosis were other possible explanations for the advanced‐stage disease observed among younger patients.^28^, ^29^

The 5‐year survival increased by 41.6% and 45.9% among younger and older GC patients, respectively, which correlates with the increasing trends of pTNM stages I and II diagnosis, surgery, and multimodality treatment. In China, GC survival trends may be attributed to an improvement in the quality of clinical services, including better access to primary healthcare, greater availability of diagnostic facilities, and more effective treatment.^4^ Since 2015, screening and early detection programs for GC have expanded to 31 provinces in China.^30^ Fortunately, the GC detection rate has increased steadily as a result of the large‐scale application of upper gastrointestinal endoscopy.^31^ Widespread decision making by multi‐disciplinary teams (MDT)^32^ and the emergence of individual multimodal therapies^33^ have also helped to improve GC prognosis.

There is still disagreement about the survival outcomes of younger patients. Some studies indicate that younger patients have worse outcomes,^14^, ^15^ while others have shown no age differences in survival rates.^12^, ^13^ Findings from the current study indicated that younger patients with pTNM stage III had a worse prognosis than older patients, while there was no age‐specific difference in the prognosis of patients with other GC stages. Thus, it is possible that differences in prognosis between the younger and older groups are related to cancer stage distribution. The higher proportion of younger patients receiving multimodality treatment may reflect a better tolerance for GC, while more advanced stage and aggressive tumors may contribute to poorer survival outcomes in this subpopulation.^14^ Reduced organ function and an increased risk of comorbidities may also impact age‐specific GC prognosis.^34^ In addition, while the genomic profiles of younger patients are not yet known, a strong association between particular gene mutations and GC prognosis has been shown in different age groups.^16^, ^17^

The present study showed differences in the prevalence of several gene mutations between younger and older GC patients, indicating that there is a specific mutational spectrum across age groups. A higher prevalence of mutations in *LRP1B,* a unique low‐density lipoprotein receptor (LDLR) that binds and internalizes ligands, was identified in older patients.^35^ The function of *LRP1B* in carcinogenesis remains controversial.^36^, ^37^, ^38^, ^39^ Several studies have indicated that *LRP1B* is a tumor suppressor and show that impaired *LRP1B* expression promotes carcinoma cell proliferation, migration, and invasion.^37^, ^38^ However, the latest update from the Network of Cancer Genes (NCG 6.0) listed *LRP1B* as a potentially false‐positive tumor suppressor.^38^ Zhou et al.^39^ demonstrated that *LRP1B* was also a recurrently mutated driver gene that is strongly associated with the development of GC. Further analysis is required to investigate the pathobiological property of *LRP1B* in GC.

Older patients in this study also had a higher likelihood of *GNAS* mutations than younger patients. *GNAS* is situated on chromosome 20q13.3 and encodes the alpha subunit of the stimulatory G‐protein.^40^ One study has shown that *GNAS* mutations activate the Wnt and ERK1/2 MAPK pathways through the autonomous synthesis of cyclic adenosine monophosphate (cAMP) and promote intestinal tumorigenesis.^41^ Additional studies have shown that *GNAS* mutations occur frequently in gastric adenocarcinoma.^42^, ^43^ Consistent with the current study, Esser et al.^40^ found that *GNAS* expression was more prevalent in well‐ and moderately differentiated GC, which potentially correlated with older age. While the underlying mechanism remains unclear, *GNAS* mutations may affect the progression of GC through activation of protein kinase A (PKA), MAPK, and Wnt signaling.^44^

As shown previously,^45^, ^46^ the current study found that older patients had a higher prevalence of *APC* mutations than younger patients. Long considered a canonical tumor suppressor, *APC* can inhibit excessive tumor cell proliferation by regulating Wnt signaling.^47^ *APC* mutation was common in GC, particularly in well‐differentiated adenocarcinoma.^48^, ^49^ Zhan et al.^50^ proposed that a strong synergy between *APC* alterations and *MEK* inhibitors enhances the signaling output of the Wnt cascades. Gerner et al.^51^ found that mutant *APC* was associated with elevated nitric oxide synthase 2 and the dysregulation of polyamine metabolism. These data suggest that *APC* mutations may contribute to GC oncogenesis. In addition, a recent study demonstrated a significant association between high *APC* expression and poor GC prognosis.^52^ This could be the result of disrupted Wnt cascades, metabolic dysregulation, or another unknown mechanism.

*KMT2D* was among the most frequently mutated genes in different types of cancer, including GC.^53^, ^54^, ^55^, ^56^ By encoding histone methyltransferase, *KMT2D* is critically involved in the epigenetic and transcriptional regulation of certain tumor‐associated genes.^55^, ^56^, ^57^, ^58^, ^59^ Lv et al.^55^ suggested that *KMT2D* may induce prostate carcinogenesis and metastasis by activating *LIFR* and *KLF4*. Xiong et al.^56^ later confirmed that *KMT2D* might play a role in cell proliferation and apoptosis during GC by down‐regulating *PTEN* and up‐regulating *LIFR* and *KLF4*. However, recent studies have also found that *KMT2D* can inhibit the development of medulloblastoma,^57^ lung tumors,^58^ and lymphoma,^59^ by activating particular pro‐apoptotic genes or repressing genes related to cell growth and survival. These results suggest that *KMT2D* may have distinct functions and biological effects in different types of cancer. Several studies have also indicated that *KMT2D* mutations were substantially higher in older patients than younger patients,^60^, ^61^ a finding also shown in the current study. More research is necessary to elucidate the underlying function of *KMT2D* in GC patients of different ages.

This study has several strengths. First, clinicopathological characteristics and survival trends of younger GC patients diagnosed from 1998 to 2018 were comprehensively described using NCCGCDB data and might serve as a reference for a large population‐based study in China. Second, this study is the first to show distinct mutations to particular genes, including *LRP1B, GNAS, APC, and KMT2D*, among younger and older patients, which may in part explain age‐specific differences in GC progression and survival at the molecular level. A major limitation of this study was the insufficient sequencing sample size, particularly for pTNM subtype analysis. As a result, the possibility might not be ruled out for some of the significant findings. In addition, there are likely to be regional and racial disparities among younger patients from China and western counties that could affect the clinicopathological and molecular properties of GC. Thus, some findings from the TCGA and MSKCC databases may not be strongly generalizable to China, and the high‐volume sequencing analysis using data from younger Chinese GC patients may require further evaluation. Finally, some variables, including tumor markers, neoadjuvant therapy, and adjuvant therapy, will need to be compared between younger and older patients.

In summary, this study found a stable proportion of young GC patients in the China National Cancer Center database and showed a significant improvement in their survival rate from 1998 to 2018. Multivariate analysis suggested that younger patients with pTNM stage III had poorer survival outcomes than older patients, while there were no age‐specific differences in survival for patients with other tumor stages. Distinct genetic alterations were further identified in younger patients, thus improving the option for precise and personalized treatment for this subpopulation. Additional large‐scale studies are warranted to investigate other molecular characteristics and related mechanisms among younger GC patients.

## CONFLICT OF INTEREST

All authors have completed the ICMJE uniform disclosure form (available at http://dx.doi.org/10.21037/atm‐20‐2554a). The authors have no conflict of interest to declare.

## AUTHORS' CONTRIBUTIONS

Yingtai Chen and Dongbing Zhao were involved in conception and design. Dongbing Zhao, Yingtai Chen, and Yawei Zhang were involved in administrative support. Penghui Niu, Huang Huang, and Lulu Zhao were involved in provision of study materials or patients Tongbo Wang, Xiaojie Zhang, and Wanqing Wang were involved in collection and assembly of data. Lulu Zhao, Penghui Niu, and Chunguang Guo were involved in data analysis and interpretation. All authors were involved in manuscript writing and final approval of manuscript.

## ETHICAL STATEMENT

The authors are accountable for all aspects of the work in ensuring that questions related to the accuracy or integrity of any part of the work are appropriately investigated and resolved. This study was approved by the ethics committee of National Cancer Center/National Clinical Research Center for Cancer/Cancer Hospital, Chinese Academy of Medical Sciences and Peking Union Medical College (No. 17–156/1412).

## Supporting information


Figure S1

Figure S2

Table S1
Table S2

## Data Availability

All data generated during this study are included in this published article and its supplementary files.
