# Supplementary figures and images for: Clinicopathological characteristics, survival outcomes, and genetic alterations of younger patients with gastric cancer: Results from the China National Cancer Center and cBioPortal datasets
Source: Cancer Med. 2022 Mar 22;11(16):3057–73. doi: 10.1002/cam4.4669 (PMC9385592; doi:10.1002/cam4.4669)

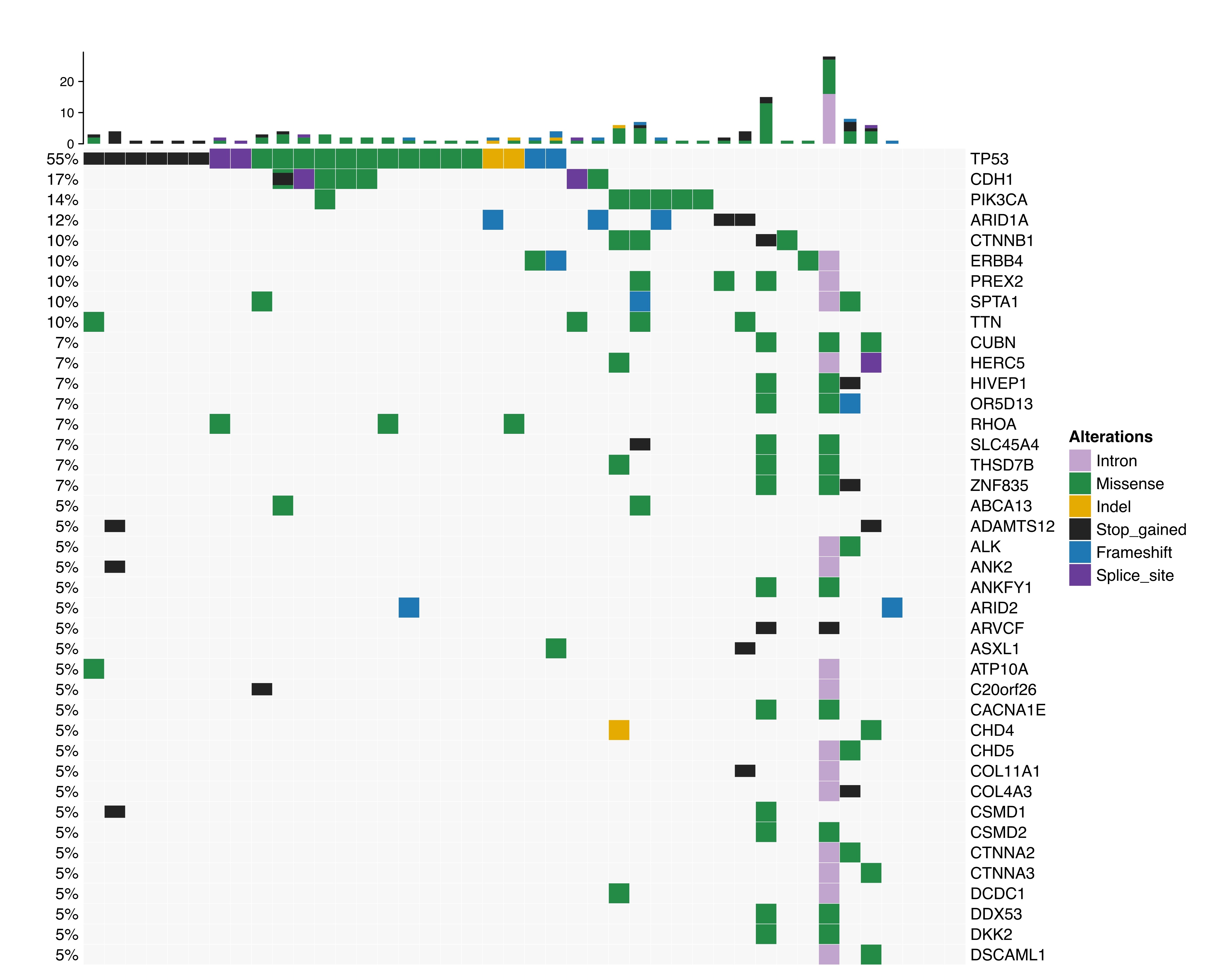

Supplement: Supplementary file 1 — Figure S1 [file CAM4-11-3057-s001.jpg]

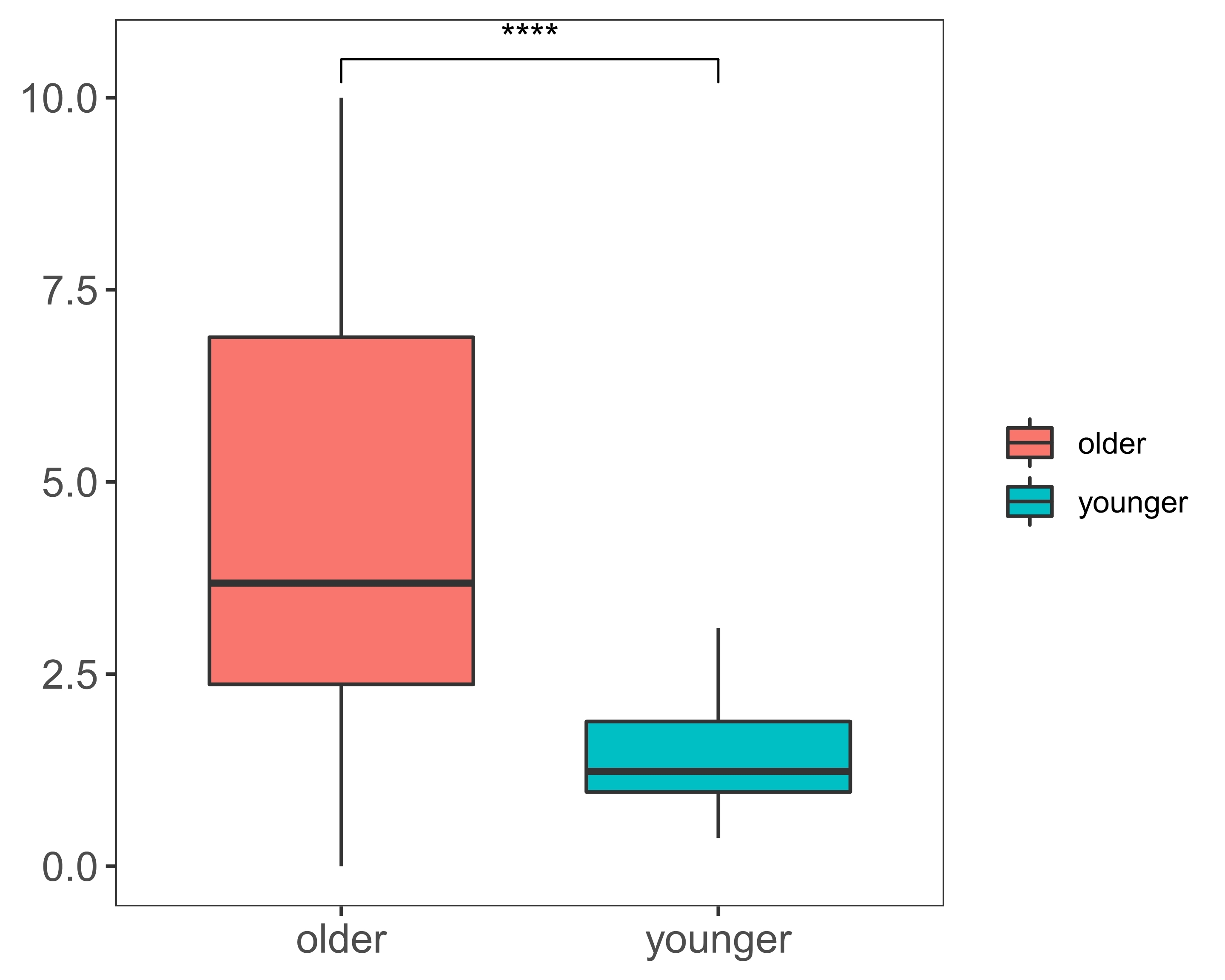

Supplement: Supplementary file 2 — Figure S2 [file CAM4-11-3057-s002.jpg]
