# Supplementary material for: Clinicopathological characteristics, survival outcomes, and genetic alterations of younger patients with gastric cancer: Results from the China National Cancer Center and cBioPortal datasets
Source: Cancer Med. 2022 Mar 22;11(16):3057–73. doi: 10.1002/cam4.4669 (PMC9385592; doi:10.1002/cam4.4669)
Supplement: Supplementary file 3 — Table S1 Table S2 [file CAM4-11-3057-s003.docx]

**Table S1. Multivariate survival analysis of predictors associated with overall survival by age group (bidirectional cohort 1998–2018)**

| **Prognostic Factors** | | Multivariate anlaysis (Younger group) | | |  | Multivariate anlaysis (Older group) | | |
| --- | --- | --- | --- | --- | --- | --- | --- | --- |
|  |  | **Hazard ratio** | **95% CI** | P Value |  | **Hazard ratio** | **95% CI** | P Value |
| **Period of diagnosis** | |  |  |  |  |  |  |  |
|  | Period 1 (1998-2003) | 1.00 |  |  |  | 1.00 |  |  |
|  | Period 2 (2004-2008) | 0.63 | 0.39-1.04 | 0.073 |  | 0.56 | 0.49-0.63 | <0.0001 |
|  | Period 3 (2009-2013) | 0.73 | 0.45-1.20 | 0.22 |  | 0.60 | 0.53-0.69 | <0.0001 |
|  | Period 4 (2014-2018) | 0.23 | 0.12-0.41 | <0.0001 |  | 0.24 | 0.20-0.28 | <0.0001 |
| **Drinking history** | |  |  |  |  |  |  |  |
|  | No |  |  |  |  | 1.00 |  |  |
|  | Yes |  |  |  |  | 0.93 | 0.86-0.99 | 0.030 |
|  | Unknown |  |  |  |  | 0.82 | 0.65-1.02 | 0.078 |
| BMI (kg/m2) at diagnosis | |  |  |  |  |  |  |  |
|  | <18.5 |  |  |  |  | 1.06 | 0.93-1.21 | 0.37 |
|  | 18.5-22.9 |  |  |  |  | 1.00 |  |  |
|  | 23-27.4 |  |  |  |  | 0.94 | 0.87-1.01 | 0.093 |
|  | ≥27.5 |  |  |  |  | 0.94 | 0.84-1.04 | 0.24 |
|  | Unknown |  |  |  |  | 1.15 | 0.97-1.37 | 0.11 |
| **Weight loss** | |  |  |  |  |  |  |  |
| **Prognostic Factors** | | Multivariate anlaysis (Younger group) | | |  | Multivariate anlaysis (Older group) | | |
|  |  | **Hazard ratio** | **95% CI** | P Value |  | **Hazard ratio** | **95% CI** | P Value |
|  | None |  |  |  |  | 1.00 |  |  |
|  | <10% |  |  |  |  | 1.06 | 0.98-1.15 | 0.14 |
|  | ≥10% |  |  |  |  | 1.23 | 1.11-1.36 | <0.0001 |
|  | Unknown |  |  |  |  | 1.08 | 0.98-1.20 | 0.13 |
| H. pylori infection |  |  |  |  |  |  |  |  |
|  | Negative |  |  |  |  | 1.00 |  |  |
|  | Positive |  |  |  |  | 1.35 | 1.07-1.70 | 0.012 |
|  | Unknown |  |  |  |  | 1.61 | 1.34-1.93 | <0.0001 |
| **Primary tumor location** | |  |  |  |  |  |  |  |
|  | Proximal | 1.00 |  |  |  | 1.00 |  |  |
|  | Distal | 0.60 | 0.44-0.81 | 0.0007 |  | 0.95 | 0.88-1.01 | 0.11 |
|  | Overlapping lesions | 0.79 | 0.46-1.36 | 0.39 |  | 1.11 | 0.96-1.29 | 0.16 |
|  | Unknown | 0.39 | 0.21-0.75 | 0.0045 |  | 0.68 | 0.55-0.85 | 0.0007 |
| **Lauren classification** | |  |  |  |  |  |  |  |
|  | Intestinal | 1.00 |  |  |  | 1.00 |  |  |
|  | Diffuse | 1.70 | 0.51-5.66 | 0.38 |  | 1.18 | 0.99-1.40 | 0.061 |
|  | Mixed | 1.43 | 0.37-5.44 | 0.60 |  | 0.97 | 0.81-1.16 | 0.75 |
| **Prognostic Factors** | | Multivariate anlaysis (Younger group) | | |  | Multivariate anlaysis (Older group) | | |
|  |  | **Hazard ratio** | **95% CI** | P Value |  | **Hazard ratio** | **95% CI** | P Value |
|  | Unknown | 2.62 | 0.81-8.52 | 0.11 |  | 1.05 | 0.89-1.23 | 0.57 |
| **Borrmann classification** | |  |  |  |  |  |  |  |
|  | Borrmann I | 1.00 |  |  |  | 1.00 |  |  |
|  | Borrmann II | 0.60 | 0.35-1.01 | 0.055 |  | 0.92 | 0.80-1.04 | 0.19 |
|  | Borrmann III | 0.95 | 0.55-1.64 | 0.84 |  | 0.97 | 0.84-1.12 | 0.66 |
|  | Borrmann IV | 1.67 | 0.93-2.98 | 0.084 |  | 1.36 | 1.15-1.61 | 0.0003 |
|  | Mixed | 0.60 | 0.08-4.71 | 0.63 |  | 1.01 | 0.59-1.73 | 0.90 |
|  | Unknown | 0.72 | 0.43-1.22 | 0.22 |  | 1.09 | 0.95-1.25 | 0.22 |
| **Linitis plastica** | |  |  |  |  |  |  |  |
|  | No | 1.00 |  |  |  | 1.00 |  |  |
|  | Yes | 1.93 | 1.09-3.41 | 0.024 |  | 1.18 | 0.90-1.56 | 0.23 |
|  | Unknown | 0.75 | 0.44-1.29 | 0.30 |  | 0.85 | 0.72-1.01 | 0.064 |
| **Differentiation** | |  |  |  |  |  |  |  |
|  | Well |  |  |  |  | 1.00 |  |  |
|  | Moderate |  |  |  |  | 1.48 | 0.96-2.29 | 0.079 |
|  | Poor |  |  |  |  | 1.59 | 1.03-2.47 | 0.038 |
|  | Undifferentiated |  |  |  |  | 0.000 | 0.000-999.999 | 0.90 |
| **Prognostic Factors** | | Multivariate anlaysis (Younger group) | | |  | Multivariate anlaysis (Older group) | | |
|  |  | **Hazard ratio** | **95% CI** | P Value |  | **Hazard ratio** | **95% CI** | P Value |
|  | Unknown |  |  |  |  | 1.64 | 1.04-2.59 | 0.033 |
| **HER2 score** | |  |  |  |  |  |  |  |
|  | 0 (-) |  |  |  |  | 1.00 |  |  |
|  | 1 (+) |  |  |  |  | 0.93 | 0.82-1.07 | 0.31 |
|  | 2 (++) |  |  |  |  | 0.88 | 0.73-1.06 | 0.19 |
|  | 3 (+++) |  |  |  |  | 1.05 | 0.84-1.32 | 0.64 |
|  | Unknown |  |  |  |  | 1.20 | 1.05-1.38 | 0.0066 |
| **Pathologic T-stage** | |  |  |  |  |  |  |  |
|  | T0+Tis |  |  |  |  | 1.38 | 0.19-10.14 | 0.75 |
|  | T1 |  |  |  |  | 1.00 |  |  |
|  | T2 |  |  |  |  | 1.40 | 1.04-1.91 | 0.029 |
|  | T3 |  |  |  |  | 2.03 | 1.45-2.83 | <0.0001 |
|  | T4 |  |  |  |  | 2.50 | 1.78-3.52 | <0.0001 |
|  | TX |  |  |  |  | 2.48 | 1.70-3.62 | <0.0001 |
| **Pathologic N-stage** | |  |  |  |  |  |  |  |
|  | N0 | 1.00 |  |  |  | 1.00 |  |  |
|  | N1 | 1.02 | 0.56-1.86 | 0.96 |  | 1.29 | 1.10-1.51 | 0.0018 |
| **Prognostic Factors** | | Multivariate anlaysis (Younger group) | | |  | Multivariate anlaysis (Older group) | | |
|  |  | **Hazard ratio** | **95% CI** | P Value |  | **Hazard ratio** | **95% CI** | P Value |
|  | N2 | 1.01 | 0.58-1.76 | 0.98 |  | 1.81 | 1.53-2.13 | <0.0001 |
|  | N3 | 1.93 | 1.16-3.22 | 0.012 |  | 2.92 | 2.49-3.44 | <0.0001 |
|  | NX | 1.72 | 0.81-3.64 | 0.16 |  | 1.68 | 1.28-2.20 | 0.0002 |
| **pTNM stage** | |  |  |  |  |  |  |  |
|  | 0 | 0.000 | 0.000-999.999 | 0.98 |  | 1.80 | 0.20-15.94 | 0.60 |
|  | I | 1.00 |  |  |  | 1.00 |  |  |
|  | II | 9.47 | 2.67-33.53 | 0.0005 |  | 1.63 | 1.18-2.25 | 0.0033 |
|  | III | 18.55 | 5.49-62.72 | <0.0001 |  | 1.72 | 1.19-2.49 | 0.0041 |
|  | IV | 62.68 | 16.80-233.78 | <0.0001 |  | 4.77 | 3.20-7.10 | <0.0001 |
|  | Unknown | 27.85 | 7.27-106.66 | <0.0001 |  | 1.66 | 1.10-2.49 | 0.015 |
| **Vascular invasion** | |  |  |  |  |  |  |  |
|  | No | 1.00 |  |  |  | 1.00 |  |  |
|  | Yes | 1.02 | 0.72-1.45 | 0.91 |  | 1.17 | 1.07-1.29 | 0.0008 |
|  | Unknown | 1.12 | 0.70-1.79 | 0.64 |  | 1.17 | 0.99-1.38 | 0.073 |
| **Therapeutic regimen** | |  |  |  |  |  |  |  |
|  | Surgery only |  |  |  |  | 1.00 |  |  |
|  | Multimodality treatment |  |  |  |  | 1.06 | 0.86-1.32 | 0.59 |
| **Prognostic Factors** | | Multivariate anlaysis (Younger group) | | |  | Multivariate anlaysis (Older group) | | |
|  |  | **Hazard ratio** | **95% CI** | P Value |  | **Hazard ratio** | **95% CI** | P Value |
|  | Unknown |  |  |  |  | 1.26 | 1.02-1.55 | 0.034 |
| **Surgical margin** | |  |  |  |  |  |  |  |
|  | Negative | 1.00 |  |  |  | 1.00 |  |  |
|  | Positive | 2.38 | 1.22-4.64 | 0.011 |  | 1.22 | 1.01-1.46 | 0.037 |
|  | Unknown | 0.89 | 0.47-1.68 | 0.72 |  | 1.33 | 1.13-1.57 | 0.0006 |

**Table S2a. Multivariate survival analysis between younger group and older group in total patients.**

| **Prognostic Factors** | | **Unadjusted** | | |  | **Adjusted** | | |
| --- | --- | --- | --- | --- | --- | --- | --- | --- |
|  |  | **HR** | **95% CI** | P **Value** |  | **HR** | **95% CI** | P **Value** |
| **Total** | |  |  |  |  |  |  |  |
|  | Younger group (<40 years) | 1.00 |  |  |  | 1.00 |  |  |
|  | Older group (≥40 years) | 0.80 | 0.71-0.90 | 0.0001 |  | 0.92 | 0.81-1.04 | 0.16 |

**Table S2b. Multivariate survival analysis between younger group and older group in pTNM I patients.**

| **Prognostic Factors** | | **Unadjusted** | | |  | **Adjusted** | | |
| --- | --- | --- | --- | --- | --- | --- | --- | --- |
|  |  | **HR** | **95% CI** | P **Value** |  | **HR** | **95% CI** | P **Value** |
| **pTNM I** | |  |  |  |  |  |  |  |
|  | Younger group (<40 years) | 1.00 |  |  |  | 1.00 |  |  |
|  | Older group (≥40 years) | 3.32 | 1.06-10.45 | 0.040 |  | 2.92 | 0.90-9.43 | 0.074 |

**Table S2c. Multivariate survival analysis between younger group and older group in pTNM II patients.**

| **Prognostic Factors** | | **Unadjusted** | | |  | **Adjusted** | | |
| --- | --- | --- | --- | --- | --- | --- | --- | --- |
|  |  | **HR** | **95% CI** | P **Value** |  | **HR** | **95% CI** | P **Value** |
| **pTNM II** | |  |  |  |  |  |  |  |
|  | Younger group (<40 years) | 1.00 |  |  |  | 1.00 |  |  |
|  | Older group (≥40 years) | 0.88 | 0.53-1.45 | 0.60 |  | 1.15 | 0.69-1.94 | 0.59 |

**Table S2d. Multivariate survival analysis between younger group and older group in pTNM III patients.**

| **Prognostic Factors** | | **Unadjusted** | | |  | **Adjusted** | | |
| --- | --- | --- | --- | --- | --- | --- | --- | --- |
|  |  | **HR** | **95% CI** | P **Value** |  | **HR** | **95% CI** | P **Value** |
| **pTNM III** | |  |  |  |  |  |  |  |
|  | Younger group (<40 years) | 1.00 |  |  |  | 1.00 |  |  |
|  | Older group (≥40 years) | 0.79 | 0.67-0.95 | 0.0096 |  | 0.80 | 0.67-0.96 | 0.014 |

**Table S2e. Multivariate survival analysis between younger group and older group in pTNM IV patients.**

| **Prognostic Factors** | | **Unadjusted** | | |  | **Adjusted** | | |
| --- | --- | --- | --- | --- | --- | --- | --- | --- |
|  |  | **HR** | **95% CI** | P **Value** |  | **HR** | **95% CI** | P **Value** |
| **pTNM IV** | |  |  |  |  |  |  |  |
|  | Younger group (<40 years) | 1.00 |  |  |  | 1.00 |  |  |
|  | Older group (≥40 years) | 0.91 | 0.74-1.12 | 0.37 |  | 1.03 | 0.84-1.28 | 0.76 |

The adjusted models were builded by using stepwise selection with minimized AIC,and the covariates included in the final models were selected by the stepwise selection method, with a signifcant level for adding variables of 0.05 and a significant level of removing variables of 0.10.
